# *Trichoderma citrinoviride*: Anti-Fungal Biosurfactants Production Characteristics

**DOI:** 10.3389/fbioe.2021.778701

**Published:** 2021-11-23

**Authors:** Michał Piegza, Kamil Szura, Wojciech Łaba

**Affiliations:** Department of Biotechnology and Food Microbiology, Wroclaw University of Environmental and Life Scinces, Wrocław, Poland

**Keywords:** T. citrinoviride, biosurfactant, biocontrol, anti-fungal activities, surface, active agent

## Abstract

The mechanism of direct impact of *Trichoderma* fungi on other organisms is a multilayer process. The level of limiting the growth of other microorganisms is determined by the strain and often by the environment. Confirmation of the presence of extracellular biosurfactants in certain strains of *Trichoderma* considered as biocontrol agents was regarded as a crucial topic complementing the characterization of their interactive mechanisms. Selected strains of *T. citrinoviride* were cultured in media stimulating biosurfactant biosynthesis, optionally supplemented with lytic enzyme inducers. Results confirmed the anti-fungal properties of surface-active compounds in the tested culture fluids. Preparations that displayed high fungal growth inhibition presented marginal enzymatic activities of both chitinases and laminarinases, implying the inhibitory role of biosurfactants. Fractions from the foam of the culture fluid of the C1 strain, cultured on Saunders medium, and HL strain on MGP medium, without an additional carbon source, exhibited the most prominent ability to inhibit the growth of phytopathogens. Filamentous fungi capable of producing fungicidal compounds, including surfactants, may find applications in protecting the plants against agri-food pathogenic molds.

## Introduction

In recent years, plant protection has become the dominant field for biological control. The application of living organisms as pesticides has become a vital alternative to the standard use of their chemical equivalents. The use of traditional pesticides tends to be severely limited due to numerous undesirable effects on human health and the environment (Thomas and Willis, 1998). Some of the microorganisms inhabiting the soil, in particular the rhizosphere, displayed the ability to control the expansion of plant pathogens (Sjingh et al., 2007). The term “biological control” has been diversely defined by various biologists, and the first definitions focused on the use of predators and insect parasites to combat other organisms. In the agricultural industry, the activity limiting the development of pathogens has been noticed among many species of bacteria and filamentous fungi. The ability of biological control over microorganisms is attributed not only to the competition for nutrients or the biosynthesis of lytic enzymes (Piegza et al., 2014) but also to other compounds secreted into the medium, including those of a biosurfactant nature (Banat et al., 2000).

The genus *Trichoderma* itself was proposed for the application in biocontrol due to the fact that these fungi are effective producers of extracellular hydrolases, including chitinases and glucanases (Harman, 2006). Majority of *Trichoderma* species have been extensively evaluated in terms of both the produced compounds and their genome sequences (Schuster and Schmoll, 2010). The *Trichoderma citrinoviride* C1 strain, formerly known as *T. hamatum* C1, has been the target of research on the directional use in the protection of plants against pathogens for many years. The beginning of this type of experiments dates back to the 1980s, and the results indicated the antagonistic behavior of *T. hamatum* in relation to other species of filamentous fungi (Chet et al., 1981). In subsequent years, the research focused mainly on determining the ability of this strain to produce lytic enzymes (CWDE—cell wall degrading enzymes), predominantly chitinases and *β*-1,3-glucanases, causing the degradation of pathogen cell walls as the main mechanisms of interaction with fungal pathogens (Witkowska and May 2002; Witkowska et al., 2009; Piegza et al., 2015; Piegza et al., 2016). It is worth to emphasize that *T. citrinoviride* C1 can facilitate efficient biotransformation, e.g., cortexolone to 1-dehydrocortexolone through 1-dehydrogenation (Bartmańska and Dmochowska-Gładysz, 2007). Recent studies have also indicated the mycoparasite activity of this species against the pathogens of the ginseng plant (*Panax ginseng*). The inhibitory effect concerning *Botrytis cinerea* is probably due to its ability to inhibit the expression of *B. cinerea* genes responsible for growth and virulence. On the other hand, stimulating the expression of ginseng genes is related to the response to pathogens, and the production of phytohormones and extracellular enzymes that degrade the pathogen cell walls is also considered (Park et al., 2019). *Trichoderma citrinoviride* exhibits a potential towards biological control as its high larvicidal and ovicidal activity has been verified against the parasitic cotton root nematode *Meloidogyne incognita* (Fan et al., 2020). Moreover, there is available reference that confirms the ability of *T. citrinoviride* isolated from rotting algae to inhibit and inactivate the toxic cyanobacteria *Microcystis aeruginosa* (Mohamed et al., 2014). The complexity of biocontrol mechanisms means that the discovery of any additional factor that may affect the correlation between organisms inspires thorough study. Such a factor was the confirmation that *T. citrinoviride* strains are capable of biosurfactants biosynthesis (Piegza et al., 2021).

The antibiotic activity of biosurfactants produced by microorganisms may play the role in competition, pathogenesis, and self-protection. At the same time, however, deepening knowledge of their chemical structure and mode of action seems important for medicine or industry. Also, information on their antiprotozoal, anti-cancer, and potentially weakening biofilms is relatively sparse (Markande et al., 2021). By modifying the liquid surface tension, biosurfactants may enhance the bioavailability of exogenous compounds, such as nutrients, by increasing their absorption, and endogenous metabolites, including phenazine antibiotics, resulting in their increased biological activity. Additionally, microbial surface-active compounds may help to protect plants against pathogens (D’aes et al., 2010). Due to their amphiphilic nature, they can interact with biological membranes, which also consist essentially of amphiphilic lipid bilayers. This enables the activity of biosurfactants as cytolytic agents with a broad spectrum of activity, due to the capability of cell membrane disruption and, as a result, inactivation of bacteria, fungi, oomycetes, and neutralization of viruses (Hutchinson and Johnstone, 1993). Microbial biosurfactants are usually characterized by the specificity of action towards particular microorganisms. These differences in the activity are attributed to the structural properties of both the surfactant and the cell membrane of the affected microorganism (D’aes et al., 2010). The first experimental observations of brown spots induced on edible mushrooms by *P. tolaasii*, as a result of pore formation in cell membranes and surface-active properties of tolaasin, indicated that these compounds may be responsible for antagonistic interactions between microorganisms (Hutchinson and Johnstone, 1993; Saikia et al., 2021).

The aim of the study was to assess the ability of selected *Trichoderma citrinoviride* strains towards the production of biosurfactants that accompany lytic enzymes, with properties limiting the development of pathogenic filamentous fungi. For this purpose, the surface tension was evaluated in culture fluids, enzymatic activities were determined, and inhibitory potential was assessed against phytopathogenic fungi, in relation to the composition of culture media.

## Materials and Methods

The research involved filamentous fungi from the collection of the Department of Biotechnology and Food Microbiology of the Wrocław University of Environmental and Life Sciences: *T. citrinoviride* C1, *T. citrinoviride* HL, and *T. citrinoviride* B3 (Piegza et al., 2021).

Cultures were carried out in 250 ml Erlenmeyer flasks containing 50 ml of medium. Each tested strain was cultured in Mineral-Glucose-Peptone (MGP) (M) medium (Piegza et al., 2021) or Saunders medium (S), optionally supplemented with 6 g/L yeast biomass (dry biomass of *Saccharomyces* sp. and *Yarrowia* sp. equal) and 10 g/L mushroom biomass (dry biomass of champignon and *Fusarium* sp—4:1) that served as an inducer and a source of carbon (Mx and Sx, respectively). The cultivation was carried out for 5 days under agitation at 160 rpm, at 25°C (Advanced ADS10000, VWR, USA), and the post-culture fluids of each variant were collected.

Surface tension measurements were performed immediately after the collection of culture fluids. For this purpose, the tear-off method was used, based on the measurement of force necessary to detach the ring from the surface of the liquid (Rakowska and Porycka, 2009; Piegza et al., 2021). The measurements were performed on a Krüss K6 manual tensiometer, at room temperature.

From each culture fluid variant three fractions were analyzed: the raw culture fluid (R), foam obtained from a culture fluid (F), and the residue after foaming (FR).

Phytopathogenic fungi (*Lecanicillium lecanii*, *Fusarium sporotrichioides*, *Penicillium* spp, *Rhizopus nigricans*, *Aspergillus niger CZ*, *Paecilomyces variotii DSM 1961*, *Botrytis cinerea 5s*, *Fusarium culmorum*, *Mucor hiemalis*, *Epicoccum nigrum*, *and Fusarium poae 1*) were deep inoculated into PDA medium (Difco). The 6-mm-diameter wells were cut into each plate with a plug, and 100 µL of a specific culture fluid fraction was introduced into the wells. The test was performed for the fluids of each of the three tested *Trichoderma citrinoviride* strains, obtained in Saunders medium (S), Saunders medium enriched with yeast biomass (inductors) (Sx), MGP medium (M), and MGP medium enriched with yeast biomass (inducers) (Mx). The plates were incubated for 7 days at 4°C. After incubation, the effect of metabolites present in the culture fluids on the growth inhibition of phytopathogenic fungi was determined. For this purpose, clear zones were measured around the wells in which no fungal growth was observed. The zone of inhibition was compared with the growth of filamentous fungi around the wells of the plates where 100 µL of distilled water was introduced into the wells.

Laminarinases and chitinases activities were assayed in each fluid fraction with the DNS method, using laminarin and chitin, respectively, substrates, by running the enzymatic reaction for 30 min at 50 °C and pH 5.0. Reaction products, i.e., glucose in the case of laminarinases and glucosamine in the case of chitinases, were determined colorimetrically using dinitrosalicylic acid (Sigma) (Piegza et al., 2016), in order to determine the effect of enzymes on the inhibition of the test filamentous fungi.

Statistical analysis: One-way analysis of variance (ANOVA) was applied along with Duncan’s test to determine statistically homogeneous groups. Statistica 13 (TIBCO Software, Inc.) software was used.

## Results

The post-culture fluids of *Trichoderma citrinoviride* C1, HL, and B3 cultivated on Saunders (S, Sx) and MGP media (M, Mx) with and without yeast biomass were subjected to a series of tests to determine their ability to produce biosurfactants with fungicidal characteristics. The first measurements of surface tension were carried out in order to assess whether the metabolites present in the tested fluids exhibit surface-active properties. In all post-culture fluids, a reduction in surface tension was found (Table 1). It is worth emphasizing that in the case of C1 and B3 strains, the addition of fungal and yeast biomass did not affect the final result. Conversely, in the case of the HL strain, the Saunders medium with the addition of biomass turned out to be more effective. The obtained results indicate the possible occurrence of surface-active compounds, which allowed continuing the research in order to determine their characteristics.

**TABLE 1:** Surface tension measurements (mN/m) in post-culture fluid, (M) MGP medium, (S) Saunders medium, (Mx) MGP medium with inductors, (Sx) Saunders medium with inductors.

Trichoderma citrinoviride	Culture medium [mN/m]			
Strain	S	Sx	M	Mx
C1	32	34	37	35
HL	36	34	37,5	35
B3	32	35	38	35
H_2_O	72	72	72	72
Control	68	67	68	67

In order to determine the fungicidal properties of the obtained preparations, the diffusion test was carried out against 11 species of phytopathogenic fungi (Table 2). Majority of crude culture fluids demonstrated an intense inhibitory effect towards *Fusarium sporotrichioides* and *Paecilomyces variotii* DSM 1961. On the contrary, no clear zones were observed for in tests with *Lecanicillium lecanii* and *Epicoccum nigrum*.

**TABLE 2:** The clear zones (cm) around the wells, raw fluid (R), foam (F), and residue after foaming (FR). "-"—no inhibition, (M) MGP medium, (S) Saunders medium, (Mx) MGP medium with inducers, (Sx) Saunders medium with inducers. *E. nigrum* and *L. lecanii* did not produce inhibition zones.

Trichoderma fluid/pathogen	*F. sporotrichioides*	*Penicillium* spp.	*R. nigricans*	*A. niger* CZ	*F. culmorum*	*B. cinerea* 5s	*M. hiemalis*	*P. variotii*	*F. poae 1*
C1 S; R	-	1.9	-	-	-	-	-	0.6	-
C1 Sx; R	0.4	-	0.4	0.2	-	-	-	0.8	-
C1 M; R	0.3	-	-	-	-	-	-	0.8	-
C1 Mx; R	0.4	0.2	0.4	-	0.2	0.1	0.9	0.5	-
HL S; R	0.5	-	-	-	0.5	-	-	0.5	-
HL Sx; R	0.3	0.2	-	-	-	-	-	0.5	-
HL M; R	-	-	0.2	-	-	-	-	0.5	1.6
HL Mx; R	-	-	-	-	-	-	-	-	-
B3 S; R	0.7	0.2	0.4	0.2	0.6	-	-	-	-
B3 Sx; R	0.4	-	0.3	-	0.7	-	0.4	0.5	-
B3 M; R	0.3	-	-	-	-	-	-	0.9	-
B3 Mx; R	0.3	-	-	-	1.2	-	-	0.5	-
C1 S; F	-	2.1	-	-	-	-	-	-	-
C1 Sx; F	0.5	0.1	0.5	-	-	-	-	0.9	-
C1 M; F	0.6	0.5	0.3	0.6	1.9	0.3	0.3	1.1	-
C1 Mx; F	0.4	0.1	0.3	-	-	-	0.7	0.7	-
HL S; F	0.6	0.2	0.4	0.2	0.7	0.2	-	1	0.5
HL Sx; F	0.4	0.1	-	-	-	-	-	0.9	-
HL M; F	0.4	-	0.3	-	-	0.3	0.3	0.7	1.9
HL Mx; F	-	-	-	-	-	-	-	-	-
B3 S; F	0.5	0.2	0.4	0.1	-	-	-	-	-
B3 Sx; F	0.3	-	0.4	-	0.4	-	0.5	0.7	-
B3 M; F	0.4	-	0.4	-	-	-	-	0.8	-
B3 Mx; F	0.4	-	-	-	0.9	-	-	0.7	-
C1 S; FR	-	2.1	-	-	-	-	-	-	0.1
C1 Sx; FR	0.5	-	0.4	0.3	-	-	-	0.7	-
C1 M; FR	0.2	-	-	-	0.5	-	-	0.7	-
C1 Mx; FR	0.3	0.1	0.3	-	-	-	0.8	-	-
HL S; FR	0.5	-	-	0.1	0.5	-	-	0.7	-
HL Sx; FR	0.4	0.1	-	-	-	0.5	-	0.8	-
HL M; FR	-	-	-	-	-	-	-	0.7	-
HL Mx; FR	-	-	-	-	-	-	-	-	-
B3 S; FR	0.5	0.2	0.5	0.2	0.3	0.2	-	0.7	-
B3 Sx; FR	0.3	-	0.3	-	0.6	-	0.4	0.6	-
B3 M; FR	-	-	-	-	-	-	-	1.2	-
B3 Mx;FR	0.3	-	-	-	1.1	-	-	0.5	-

The comparison of the inhibitory effect of individual post-culture fluid fractions revealed the highest fungicidal properties of foam preparations. In the vast majority of cases, the compounds present in the F fraction induced the largest inhibition zones. Foam fractions from cultures of the C1 strain in MGP and HL in Saunders medium exhibited significant activity against the majority of the tested phytopathogens. Clear zones in plate tests occurred in 8 out of 11 species of filamentous fungi tested (Table 2).

For selected culture variants (S, M), the foam fraction was separated with ultrafiltration, on a membrane with a 10 kDa cutoff, to separate the low molecular weight biosurfactants from larger accompanying proteins, including enzymes (Table 3). The well diffusion test was repeated with 20 µL of fractions <10 kDa and 50 µL of the residual fraction <10 kDa. As a result, it turned out that low molecular weight biosurfactants derived from the foam after culturing the HL and C1 strains restricted the growth of *Fusarium* and *Aspergillus* strains. The extent of inhibition was moderate due to the use of the lowest possible amount of the preparation. Furthermore, the >10 kDa fractions of foam containing high molecular weight compounds retained their antifungal activity (Table 3).

**TABLE 3:** Clear zones diameter (cm) in inhibition test with foam fractions (F) after separation through ultrafiltration. “-”—no inhibition, (M) MGP medium, (S) Saunders medium. *E. nigrum* and *M. hiemalis* did not produce inhibition zones.

*Trichoderma* strain	Culture medium	Fraction	*R. nigricans*	*L. lecanii*	*Penicillium* spp.	*F. poae*	*F. sporotrichioides*	*A. niger* CZ
HL	S	<10 kDa	-	-	-	0.20	0.10	0.10
		>10 kDa	0.20	-	0.10	0.70	-	0.10
HL	M	<10 kDa	-	-	-	0.20	0.20	-
		>10 kDa	0.20	0.20	0.10	0.45	-	0.10
C1	M	<10 kDa	-	-	-	0.20	0.10	-
		>10 kDa	0.20	1.00	0.10	0.15	0.40	0.30
B3	S	<10 kDa	-	-	-	-	-	-
		>10 kDa	0.20	-	0.10	0.50	-	0.10

The addition of yeast and fungal biomass to cultures of *Trichoderma* strains, as an inducer of the biosynthesis of lytic enzymes, resulted in the appearance of the inhibitory effect on a greater number of species of tested fungi. However, in a few cases, supplementation caused an opposite effect and reduced the zones of inhibition.

In all tested samples, a dominant activity of laminarinases over that of chitinases was observed. As expected, higher activities were achieved generally in cultures with the addition of inducers. Moreover, it is worth noting that the biosynthesis level of laminarinases did not differ significantly depending on the producer strain.

**FIGURE 1:**
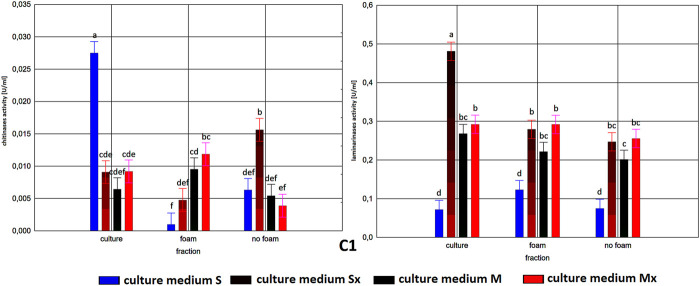
Enzymatic activities in three fractions: raw (R), foam (F), foaming residue (FR), of *Trichoderma citrinoviride* B3 strain cultivated in different culture media, after separation with foaming technique (M) MGP medium, (S) Saunders medium, (Mx) MGP medium with inductors, (Sx) Saunders medium with inductors. One-way analysis of variance (ANOVA) was applied; a … i—homogeneous groups according to Duncan’s test.

**FIGURE 2:**
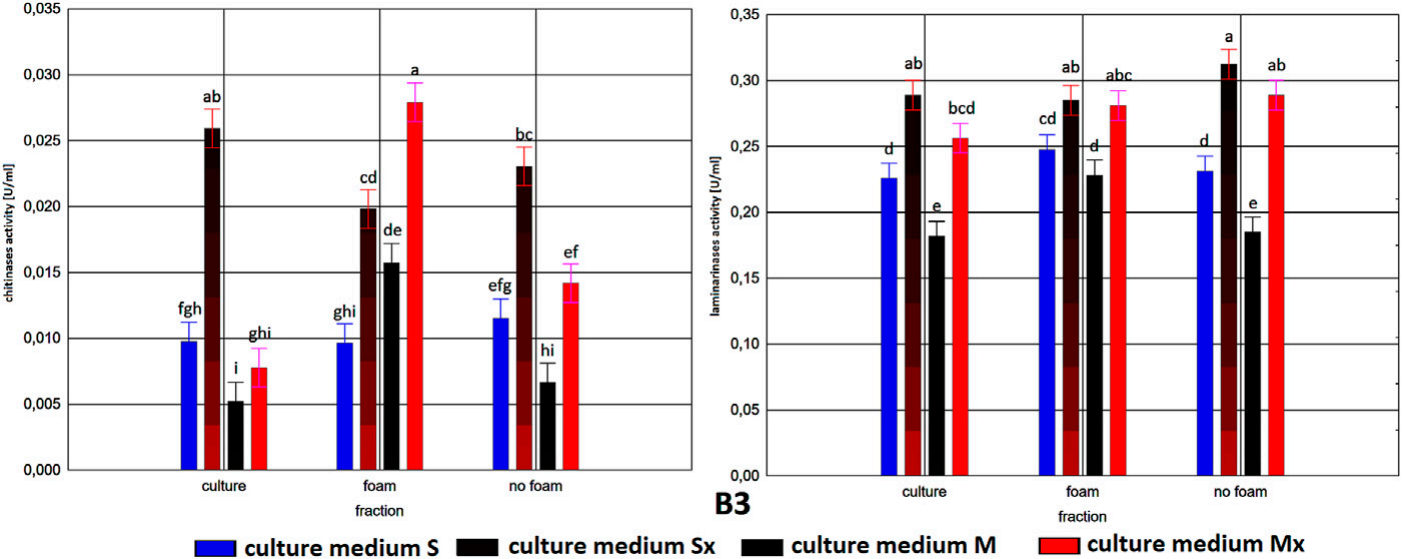
Enzymatic activities in three fractions: raw (R), foam (F), foaming residue (FR), of *Trichoderma citrinoviride* C1 strain cultivated in different culture media, after separation with foaming technique (M) MGP medium, (S) Saunders medium, (Mx) MGP medium with inductors, (Sx) Saunders medium with inductors. One-way analysis of variance (ANOVA) was applied; a … i—homogeneous groups according to Duncan’s test.

**FIGURE 3:**
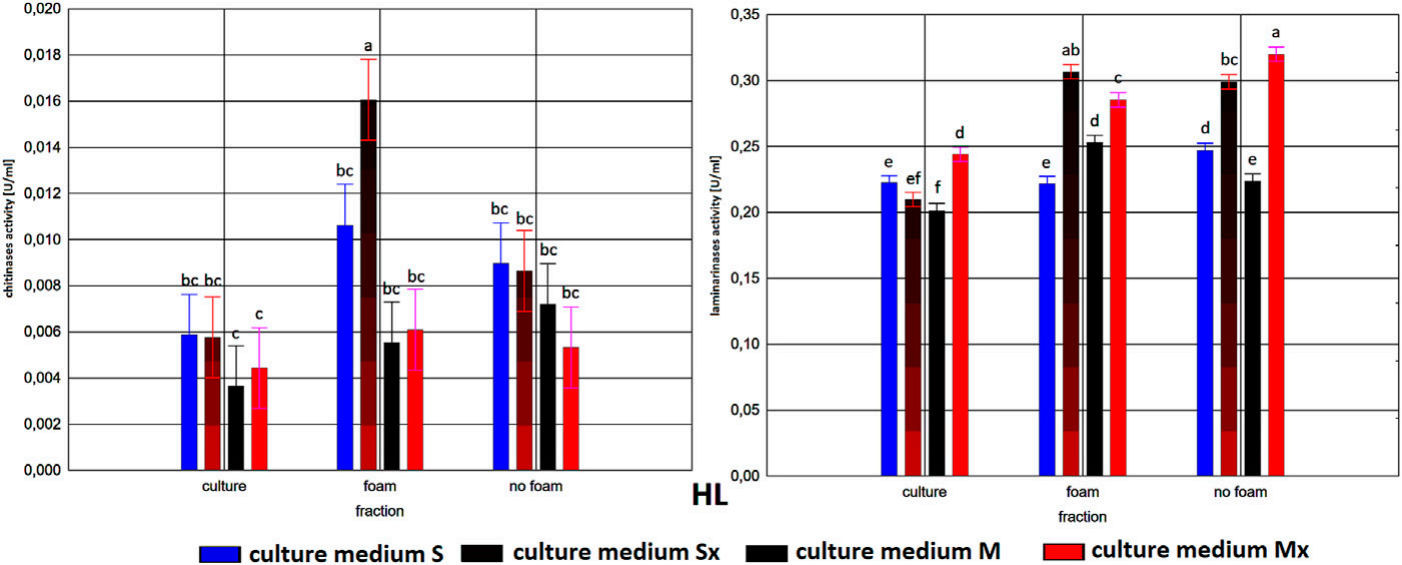
Enzymatic activities in three fractions: raw (R), foam (F), foaming residue (FR), of *Trichoderma citrinoviride* HL strain cultivated in different culture media, after separation with foaming technique (M) MGP medium, (S) Saunders medium, (Mx) MGP medium with inductors, (Sx) Saunders medium with inductors. One-way analysis of variance (ANOVA) was applied; a … i—homogeneous groups according to Duncan’s test.

The enzymatic activities were determined in three fractions of culture fluids. The highest value of laminarinases activity was 0.481 U/ml, observed in the R fraction of the C1 strain grown in Saunders medium with the addition of biomass. The following highest values of activity, at the level of ca. 0.3 U/ml, were found for the HL strain in MGP and Saunders medium, as well as the B3 strain in Saunders medium, in each case with the addition of inducing biomass. The lowest activities were recorded for the C1 strain grown in Saunders medium without biomass enrichment, for all three fractions, especially R and FR (Figures 1–3).

Comparison of culture medium variants and fractions for each separate strain allowed distinguishing Saunders medium supplemented with biomass as favorable for laminarinases biosynthesis by the C1 and B3 strain. Importantly, enzymatic activity in the raw culture fluid (R) of the B3 strain compared to both F and FR fractions remained at a similar level and also gave the C1 strain a comparable result. In the case of the HL strain, the activity of laminarinases in the foam fraction (F) from the Saunders (Sx) medium was higher than in both F and FR fractions.

The biosynthesis of chitinases in media dedicated to the biosurfactants overproduction and supplemented with the biomass was characterized by a much greater differentiation than the biosynthesis of the previously discussed enzyme. While the highest activity was recorded for the C1 strain, its biosynthesis took place on a pure Saunders medium. More importantly, its high level was neither recorded in the F nor in the FR fraction. For this strain, it was also noted that the chitinases biosynthesized on Saunders medium with inducers remained in the FR fraction, while from the MGP medium with inducers migrated to foam (F). For the B3 strain, where the highest levels of chitinase biosynthesis were recorded in Saunders medium with inducers, these activities were evenly distributed in both foaming and foaming residues (F and FR). The chitinases biosynthesis by the HL strain, as well as their activity in all three assessed fractions, with the exception of the high level of chitinase obtained in Saunders culture with inducers, was practically equal. Certainly, the level of activity, as in all the cases, depended on the volume of foam obtained and thus the volume of the residual fraction, but in order to assess the inhibitory role of biosurfactants, this was not relevant as the main object of the study (Figures 1–3).

As to the fractions of C1 and HL strains that exhibited significant clear zones in the well test or the action against many fungal species, with the tested enzymatic activities, they are characterized by a very low activity of chitinases and relatively low of laminarinases. Due to low enzymatic activities and large inhibition zones, the obtained results may indicate the presence of fungicidal biosurfactants in the post-culture fluids, which were predominantly present in the F fraction. In terms of the production of these compounds, the *T. citrinoviride* C1 and HL strains cultivated in both Saunders and MGP media, without the addition of yeast and fungal biomass, deserve special consideration.

## Discussion

Biosurfactants produced by filamentous fungi using renewable substrates (sources) become a versatile and sustainable alternative to synthetic petroleum-based surfactants. The public attention is drawn to the use of environmentally safe products obtained with “green” methods is encouraged (da Silva et al., 2008). The results obtained in the experiment allowed us to conclude that the filamentous fungi of the *Trichoderma citrinoviride* species have the ability to secrete extracellularly not only hydrolytic enzymes but also presumably biosurfactants with antimicrobial characteristics. The latter may have a direct effect on the microbial cells and additionally support the action of hydrolases.

Leaving aside the basic theories of the phenomenon of *Trichoderma* fungi involved in biocontrol processes, based mainly on CWDE, the presented results may additionally constitute an important aspect in the discussion explaining the evolutionary purpose of microbial biosurfactants biosynthesis. The considered concepts of biosurfactants role indicate the emulsifying properties and solubility improvement of nutritive compounds in the immediate surroundings, changing environmental parameters, facilitating the attachment of fungal cells to cell walls of other organisms, but also their antibiotic activity to counteract competitors in the environment (da Silva et al., 2021).

The synthesis of surfactants can occur spontaneously or as a result of induction due to the presence of lipid compounds, temperature fluctuations, pH, stirring, cellular stress, and low concentration of nitrogen in the medium. Probably, the main source of biosurfactant synthesis is carbohydrates in the culture medium. Their availability regulates the course of glycolysis and lipogenesis, whereby the hydrophilic or hydrophobic components of surfactant are synthesized (da Silva et al., 2008; Kalyani et al., 2014).

The tested culture fluids exhibited a significantly reduced surface tension, even to the level of 32 mN/m, which can be considered a substantial result, especially when compared with the values obtained for other microorganisms. For example, Mennes et al. (2017) reported the capability of *Aureobasidium thailandense* LB01 to produce biosurfactants on waste materials such as olive oil mill wastewater. After partial purification, the biosurfactants present in the culture fluid could lower the surface tension to 31.2 mN/m. According to Fechter et al. (2017), the high-strength bacterial biosurfactant is capable of reducing surface tension maximally to the level 22–25 mN/m. On the other hand, Lima et al. (2016) out of eight filamentous fungi analyzed in terms of the production of biosurfactants picked a single *Phoma* sp. strain that exhibited a promising potential. The results indicated that the surface tension of the post-culture extract was 51.03 mN/m. The ability of the culture extract to emulsify diesel fuel was also demonstrated.

The emulsifying properties are a generally well-researched phenomenon; however, it may reflect some interactions between biosurfactant molecules and microorganisms. Hence, the focus was on a simple but highly effective method of assessing this relationship. The results of the diffusion tests performed revealed a great potential to inhibit the development of pathogenic fungi concerning the proportion of surfactants present in the tested post-culture fluids. The preceding separation of the obtained post-culture fluids into P, F, and FR fractions allowed us to initially determine the inhibitory potential of the tested biosurfactants against phytopathogens, rather than their characteristics, as they were obtained in earlier studies (Piegza et al., 2021).

Thus, particularly impressive inhibition was denoted in the separated fractions of foam (F). It was the foam fractions that caused both the largest extent of inhibition and the widest range of inhibited fungal strains. The visible clear zones in the diffusion plate tests occurred in 9 out of 11 species of phytopathogens tested in the experiment. Importantly, in many trials, the diameter of the inhibition zones was ca. 1.0 cm, and the highest values reached 1.9 cm. In the report that involved diffusion tests to determine the antibacterial activity of biosurfactants clear zones of the inhibited growth of pathogenic bacteria were notably less distinct (Wieczorek et al., 2014). In most cases, their diameter was below 0.5 cm, with occasional highest values of ca. 1.1 cm. At the same time, the additional separation of foam into two fractions (above and below 10 kDa) and the well diffusion test performance allowed to put forward the thesis about the combined action of lytic enzymes and biosurfactants, visible in the preceding test involving unseparated foam fractions and presenting clearly stronger inhibition of pathogens. This type of combined effect is also observed, e.g., with a cocktail of antibiotics and a biosurfactant (Hage-Hülsmann et al., 2018).

Other studies confirm the capability of different species of the genus *Trichoderma* to produce compounds of fungicidal nature. In the study by Witkowska et al. (2009), *T. harzianum* T33 was used in an inhibitory test against pathogenic *F. poae*, while *T. citrinoviride* T2 and C1 against *F. avenaceum*. The test showed distinct zones of pathogen growth inhibition around the discs with *Trichoderma* strains. In the mentioned report and in the results from the presented study, impressive zones of inhibition of pathogen growth were obtained in the case of the *T. citrinoviride* C1 strain. This may signify the profound effectiveness of its prospective use in the protection of plants against phytopathogens. Comparable positive results of the plate tests were also observed in the trials with the use of HL and B3 strains. The number of positive results from the diffusion tests, as well as the satisfactory extent of the clear zones around the wells, may indicate the potential fungicidal characteristics of biosurfactants produced by the *T. citrinoviride* strains. The conclusions from this experiment allow us to imply that filamentous fungi of the *T. citrinoviride* species possess the ability to biosynthesize biosurfactants with anti-fungal characteristics. The abovementioned test results indicated the possibility of using *Trichoderma* fungi to produce natural surfactants, causing inhibition of other, especially pathogenic fungal species. Similar features of these compounds are described in the literature. Surfactants isolated from *Aspergillus ustus* MSF3 showed significant inhibitory activity against *Candida albicans* and gram-negative bacteria. Sophorolipids from the mutant strain *Candida bombicola* ATCC 22214 are used in the cosmetics industry due to their properties, including radical-scavenging, stimulating fibroblast metabolism, and hygroscopic properties supporting the physiological health of the skin (Bhardwaj et al., 2013). Rhamnolipids produced by few species are recognized as a plant protection against many different bacteria and fungi (Vatsa et al., 2010). The awareness that the fraction derived from the foam of *T. citrinoviride* post-culture fluids is characterized by the greatest intensity of inhibiting the development of pathogens may be of great importance in further directional research and facilitate the use of these compounds in industry. Also, the knowledge that the fraction derived from the foam of *T. citrinoviride* culture fluids is characterized by the greatest intensity of inhibitory effect against phytopathogens may be of great importance in further research and facilitate the use of these compounds in industry.

Demonstrating the complexity of Trichoderma secondary metabolites may further affirm the importance of their introduction in environmentally friendly agricultural cultivation. Because of the recent emphasis on their antimicrobial properties and plant growth promotion, biosurfactants gain exceptional significance. Surface-active compounds are already used to remediate soil of trace elements and toxic residues. However, typical surfactants of chemical origin contradict the concept of ecological neutrality. Hence, the application of microbiological surfactants could have a positive impact on halting soil degradation with simultaneous support of plant development and maintenance of natural microbial balance (Kumar et al., 2021).

The possibility of using ecological fungicides may be of great importance, in particular for the agrotechnical industry. In the era of constant struggle to replace chemical pesticides, their natural equivalents that do not pose an environmental burden and effectively inhibit the development of pathogenic fungi turn out to be a favorable research object. The properties of *T. citrinoviride* involved in this study may contribute to the broader use of this species in the search for new solutions for the biological control of plant pathogens.

## Conclusion

The complexity of processes involved in microbial coexistence, especially in the context of their competition for living space, is still a boundless field for further discoveries. Previous findings regarding *Trichoderma* fungi indicated the participation of CWDE, a broadly understood competition mechanism, or antibiosis based on peptaibols or terpenes. However, within the same species, differences in the biocontrol mechanisms were pointed out. The finding that the most active strains in this regard additionally produce biosurfactants, of which synergistic action with other compounds is confirmed in the literature, appears to be an important contribution to the understanding of the biology of *Trichoderma* fungi.

## Data Availability

The raw data supporting the conclusions of this article will be made available by the authors, without undue reservation.
